# Multiple primary cancers in the Vaud Cancer Registry, Switzerland, 1974-89.

**DOI:** 10.1038/bjc.1993.72

**Published:** 1993-02

**Authors:** F. Levi, L. Randimbison, V. C. Te, I. Rolland-Portal, S. Franceschi, C. La Vecchia

**Affiliations:** Institut universitaire de médecine sociale et préventive, Centre Hospitalier Universitaire Vaudois, Lausanne, Switzerland.

## Abstract

Data collected by the Cancer Registry of the Swiss Canton of Vaud (whose population in 1980 was about 530,000 inhabitants) were used to estimate the incidence of second metachronous primary cancers following any specific neoplasm. Among 34,615 cases of incident neoplasms registered between 1974 and 1989 and followed through integrated active follow-up to the end of 1989, for a total of 118,241 person-years at risk, there were 2,185 second primaries (1,280 males, 905 females). For both sexes, the standardised incidence ratios (SIR) were significantly elevated by about 20%. Overall significantly elevated ratios were registered for cancers of the oral cavity and pharynx (SIR = 1.6 for males, 2.0 for females), oesophagus in males (SIR = 1.5), lung in males (SIR = 1.4), skin melanoma (SIR = 1.7 for males, 1.5 for females), non-melanomatous skin cancers (SIR = 1.6 for males, 1.5 for females), female breast (SIR = 1.3), kidney (SIR = 1.5 for males, 1.9 for females), and thyroid in males (SIR = 2.4). When specific first cancer sites were considered, the SIR following a cancer of the oral cavity and pharynx was around 3 in both sexes, mainly on account of a substantial excess of second primaries of the oral cavity, oesophagus, larynx and lung. The overall SIR following laryngeal cancer was 3.0, and significant excesses were observed for oral cavity and pharynx, oesophagus and lung. After lung cancer, the overall SIR was 1.7 for males and 2.6 for females, and significantly elevated SIRs were observed for oral cavity, lung and oesophagus. Following non-melanomatous skin cancers, elevated SIRs were observed in both sexes for skin melanoma and non-melanomas. The incidence of any cancer after breast cancer was significantly elevated (SIR = 1.2), mainly on account of an elevated risk of subsequent breast cancer (SIR = 1.7). With reference to cervical cancer, there was a significant excess for any subsequent primary (SIR = 1.6), and for lung cancer (SIR = 7.8). Significantly elevated SIRs were observed for kidney following bladder cancer, and for bladder after kidney cancer. In both sexes, the incidence of cancers of any site was elevated following leukaemias (SIR = 1.7 for males, 2.5 for females), and a significant excess was registered for lung in males and non-melanomatous skin cancers in both sexes.(ABSTRACT TRUNCATED AT 400 WORDS)


*Br. J. Cancer* (1993), **67**, 391–395 © Macmillan Press Ltd., 1993

### Multiple primary cancers in the Vaud Cancer Registry, Switzerland, 1974–89

F. Levi[1], L. Randimbison[1,2], V.-C. Te[1], I. Rolland-Portal[1], S. Franceschi[3] & C. La Vecchia[2,4]

[1]*Registre vaudois des tumeurs, Institut universitaire de médecine sociale et préventive, Centre Hospitalier Universitaire Vaudois, Falaises 1, 1011 Lausanne;* [2]*Institut universitaire de médicine sociale et préventive, Bugnon 17, 1005 Lausanne, Switzerland;* [3]*Servizio di Epidemiologia, Centro di Riferimento Oncologico, Via Pedemontana Occ, 33081 Aviano (PN);* [4]*Istituto di Ricerche Farmacologiche 'Mario Negri', Via Eritrea 62, 20157 Milano, Italy.*

**Summary** Data collected by the Cancer Registry of the Swiss Canton of Vaud (whose population in 1980 was about 530,000 inhabitants) were used to estimate the incidence of second metachronous primary cancers following any specific neoplasm. Among 34, 615 cases of incident neoplasms registered between 1974 and 1989 and followed through integrated active follow-up to the end of 1989, for a total of 118,241 person-years at risk, there were 2,185 second primaries (1,280 males, 905 females). For both sexes, the standardised incidence ratios (SIR) were significantly elevated by about 20%. Overall significantly elevated ratios were registered for cancers of the oral cavity and pharynx (SIR = 1.6 for males, 2.0 for females), oesophagus in males (SIR = 1.5), lung in males (SIR = 1.4), skin melanoma (SIR = 1.7 for males, 1.5 for females), non-melanomatous skin cancers (SIR = 1.6 for males, 1.5 for females), female breast (SIR = 1.3), kidney (SIR = 1.5 for males, 1.9 for females), and thyroid in males (SIR = 2.4). When specific first cancer sites were considered, the SIR following a cancer of the oral cavity and pharynx was around 3 in both sexes, mainly on account of a substantial excess of second primaries of the oral cavity, oesophagus, larynx and lung. The overall SIR following laryngeal cancer was 3.0, and significant excesses were observed for oral cavity and pharynx, oesophagus and lung. After lung cancer, the overall SIR was 1.7 for males and 2.6 for females, and significantly elevated SIRs were observed for oral cavity, lung and oesophagus. Following non-melanomatous skin cancers, elevated SIRs were observed in both sexes for skin melanoma and non-melanomas. The incidence of any cancer after breast cancer was significantly elevated (SIR = 1.2), mainly on account of an elevated risk of subsequent breast cancer (SIR = 1.7). With reference to cervical cancer, there was a significant excess for any subsequent primary (SIR = 1.6), and for lung cancer (SIR = 7.8). Significantly elevated SIRs were observed for kidney following bladder cancer, and for bladder after kidney cancer. In both sexes, the incidence of cancers of any site was elevated following leukaemias (SIR = 1.7 for males, 2.5 for females), and a significant excess was registered for lung in males and non-melanomatous skin cancers in both sexes. Some of the associations observed can be related to common risk factor exposure, such as tobacco (and alcohol) for multiple primaries of the upper digestive and respiratory tract, or tobacco for the excess of lung cancer following bladder and probably cervical cancer. However, the overall excess risk of a second primary cancer is relatively limited, and at least in part attributable to increased surveillance.

There are several reports of multiple primary cancers, mostly based on clinical or autopsy series (Berg, 1967, 1970; Schottenfeld, 1969, 1971; Watanabe *et al.*, 1984). These have suggested elevated rates due to common aetiological factors (e.g. for tobacco or diet-related cancers), or the consequences of treatment (e.g., high leukaemia risk following treatment of Hodgkin's disease, cervical, ovarian cancer, kidney or other neoplasms) (Boice *et al.*, 1985*a*; Boivin *et al.*, 1986; Kaldor *et al.*, 1987, 1990*a*, 1990*b*).

There are, however, only a few published series including systematic overviews of multiple primary cancers from cancer registration schemes, including one from Denmark (1943–80; Storm *et al.*, 1985), one from Connecticut (1935–82; Curtis *et al.*, 1985; see also US Department of Health and Human Services, 1985), and one from Finland (1953–79; Teppo *et al.*, 1985). Although several patterns of associations were similar in these studies, there were various quantitative differences: for instance, in Connecticut cancer patients had an overall 31% increased risk of developing a subsequent cancer, whereas no excess risk was observed in Denmark or Finland.

Differences in study methodology may explain at least part of these apparent discrepancies, but it is also possible that the impact of common genetic susceptibility and environmental factors, such as aetiologic aspects and/or consequences of cancer treatment are different in various populations (Boice *et al.*, 1985*b*). Thus, to provide further quantitative information on the issue, we present in this paper a summary overview of multiple primary cancers registered in the Swiss Vaud Cancer Registry from 1974 to 1989.

Correspondence: F. Levi.
Received 19 May 1992; and in revised form 4 September 1992.

### Materials and methods

The data considered for the present analysis were derived from the Vaud Cancer Registry dataset, which includes information concerning incident cases of malignant neoplasms occurring in the canton (about 530,000 inhabitants in 1980) (Levi, 1982, 1987).

Notification is based on a voluntary agreement between the recording medical institutions of the canton and the registry. All hospitals, pathological laboratories and most practitioners are asked to report all new or past cases of cancer. The main source of notification is the Cantonal University Pathological Department of Lausanne which performs the majority of histological examinations for the population covered by the Registry.

The main information available from the register comprises sociodemographic characteristics of the patient (i.e. age, sex), primary site and histological type of the tumour according to the standard International Classification of Diseases for Oncology (ICD-O), and time of diagnostic confirmation (histological or clinical diagnosis).

Passive and active follow-up is recorded and each subsequent item of information concerning an already registered case is used to complete the record of that patient. Information coming from death certificate is added to the morbidity file. Cases known only through the death certificate ('Death Certificate Only' cases (DCO)) contribute less than 5% of the average number of new cases registered per year.

The registry is tumour based and multiple primaries occurring in the same person are included separately whenever morphologically different (according to the pathological report) or occurring at different anatomical sites (defined at the third-digit level of the ICD-O topographical code). However, multiple non-melanomatous skin tumours are classified by the site of the first recognised tumour of the same morphological type.

After exclusion of 1,416 (4%) cases diagnosed at autopsy or by death certificate alone (811 and 605 among males and females, respectively), the present series comprises a total of 34, 615 cases of first diagnosed cancer primaries (18,001 and 16,614 among males and females, respectively) registered from 1974 to 1989 in the population of the Swiss canton of Vaud.

Among these cases of incident neoplasms, followed-up to the end of 1989 for a total of 118,241 person-years at risk, there were 2,185 (1,280 males, 905 females) metachronous (i.e. diagnosed at least two months after the first primary) second primaries. Tumours occurring synchronously, accounting for about 25% of all the pairs of primaries, were excluded from the present analysis. Histological confirmation was performed in 93% of the first as well as second primaries considered.

Calculation of expected numbers was based on sex-, age-, and calendar year-specific incidence rates multiplied by the observed number of person-years at risk. The end of the follow-up was determined by a second primary, death, emigration or the end of the study period at 31 December 1989. The significance of the observed/expected ratios (standardised incidence ratio, SIR) was based on the exact Poisson distribution applied to the observed numbers (Breslow & Day, 1987).

### Results

Table I gives the total number of second primary cancers following any first primary site, and the corresponding SIR for each selected cancer site and sex. Over the 14-year period considered a total of 2,185 second primaries were registered in the Vaud Cancer Registry (1,280 males, 905 females). For both sexes, these figures were significantly elevated by about 20% (SIR 1.2 in both sexes). When specific cancer sites were considered, significantly elevated rates were registered for cancers of the oral cavity and pharynx in both sexes (SIR = 1.6 for males, 2.0 for females), oesophagus and lung in males (SIR = 1.5 and 1.4, respectively), skin melanoma

**Table I** Subsequent primary malignant tumour at selected sites, and corresponding standardised incidence ratios (SIR), following any first primary site in males and females. Vaud, Switzerland, 1974–1989

| *Site of second primary tumour* | *Males* Observed | *Males* SIR | *Females* Observed | *Females* SIR |
|---|---|---|---|---|
| Oral cavity and pharynx | 59 | 1.6[b] | 20 | 2.0[b] |
| Oesophagus | 37 | 1.5[b] | 8 | 0.9 |
| Stomach | 37 | 0.8 | 17 | 0.7 |
| Colorectum | 120 | 1.1 | 111 | 1.2 |
| Liver | 16 | 0.9 | 2 | 0.5 |
| Gall bladder | 8 | 1.1 | 12 | 0.9 |
| Pancreas | 18 | 0.7 | 18 | 0.9 |
| Larynx | 15 | 1.0 | 1 | 0.7 |
| Lung | 215 | 1.4[c] | 27 | 1.1 |
| Bone | – | – | 1 | 1.9 |
| Connective and soft tissue | 6 | 2.2 | 8 | 2.0 |
| Skin melanoma | 24 | 1.7[b] | 23 | 1.5 |
| Skin non-melanoma | 363 | 1.6[c] | 262 | 1.5[c] |
| Breast | 3 | 1.3 | 208 | 1.3[c] |
| Cervix uteri | | | 9 | 0.5 |
| Corpus uteri | | | 31 | 0.9 |
| Ovary | | | 19 | 0.8 |
| Prostate | 161 | 1.0 | | |
| Testis | 6 | 2.2 | | |
| Bladder | 54 | 1.2 | 14 | 1.2 |
| Kidney | 27 | 1.5[a] | 21 | 1.9[b] |
| Brain and nerves | 4 | 0.6 | 3 | 0.6 |
| Thyroid | 6 | 2.4[a] | 11 | 1.7 |
| Lymphomas | 32 | 1.2 | 23 | 1.2 |
| Hodgkin's disease | 2 | 1.0 | 3 | 1.5 |
| Multiple myelomas | 7 | 0.7 | 1 | 0.1 |
| Leukaemias | 23 | 1.2 | 12 | 0.9 |
| All sites | 1280 | 1.2[c] | 905 | 1.2[c] |
| All sites, excluding skin non-melanoma | 917 | 1.2[c] | 643 | 1.1[b] |

[a]: $P<0.05$; [b]: $P<0.01$; [c]: $P<0.001$.

**Table II** Subsequent primary malignant tumours at selected sites, and corresponding standardised incidence ratios (SIR), following selected cancers for males and females in Vaud, Switzerland, 1974–1989

| *Site of second primary tumour* | *Males* Observed | *Males* SIR | *Females* Observed | *Females* SIR |
|---|---|---|---|---|
| *Site of first cancer*: Oral cavity and pharynx | | | | |
| Any site* | 95 | 3.4[c] | 20 | 2.9[c] |
| Oral cavity and pharynx | 21 | 13.3[c] | 9 | 77.4[c] |
| Oesophagus | 17 | 19.7[c] | 2 | 20.7[b] |
| Larynx | 4 | 6.6[b] | 0 | – |
| Lung | 37 | 6.4[c] | 3 | 10.5[b] |
| *Site of first cancer*: Oesophagus | | | | |
| Any site* | 7 | 1.1 | 2 | 1.3 |
| Oral cavity and pharynx | 3 | 9.6[b] | 0 | – |
| Lung | 3 | 2.4 | 0 | – |
| *Site of first cancer*: Larynx | | | | |
| Any site* | 55 | 3.0[c] | 2 | 1.5 |
| Oral cavity and pharynx | 8 | 7.6[c] | 1 | 41.6[a] |
| Oesophagus | 3 | 5.3[a] | 0 | – |
| Lung | 22 | 5.7[c] | 1 | 16.5 |
| *Site of first cancer*: Lung | | | | |
| Any site* | 72 | 1.7[c] | 12 | 2.6[b] |
| Oral cavity and pharynx | 7 | 2.8[a] | 3 | 36.1[c] |
| Oesophagus | 4 | 3.1[a] | 0 | – |
| Lung | 16 | 1.7[a] | 2 | 9.6[a] |
| *Site of first cancer*: Stomach | | | | |
| Any site* | 15 | 0.8 | 2 | 0.2 |
| Skin, non-melanoma | 6 | 1.4 | 18 | 4.1[c] |
| *Site of first cancer*: Colorectum | | | | |
| Any site* | 90 | 1.2 | 63 | 1.1 |
| Colorectum | 21 | 1.9[b] | 14 | 1.5 |
| Prostate | 25 | 1.5[a] | | |
| *Site of first cancer*: Skin melanoma | | | | |
| Any site* | 16 | 1.1 | 13 | 0.8 |
| Colorectum | 5 | 2.6[a] | 1 | 0.4 |
| Skin non-melanoma | 6 | 1.4 | 18 | 4.1[c] |
| Prostate | 5 | 2.0 | | |
| *Site of first cancer*: Skin non-melanoma | | | | |
| Any site* | 336 | 1.0 | 189 | 1.0 |
| Skin melanoma | 18 | 3.0[c] | 12 | 2.3[b] |
| Skin non-melanoma | 223 | 2.2[c] | 112 | 1.9[c] |
| *Site of first cancer*: Breast | | | | |
| Any site* | | | 187 | 1.2[b] |
| Colorectum | | | 26 | 1.1 |
| Pancreas | | | 7 | 1.4 |
| Breast | | | 75 | 1.7[c] |
| Corpus uteri | | | 12 | 1.2 |
| Ovary | | | 6 | 0.9 |
| Thyroid | | | 4 | 2.2 |
| *Site of first cancer*: Cervix uteri | | | | |
| Any site* | | | 33 | 1.6[b] |
| Colorectum | | | 4 | 1.4 |
| Lung | | | 7 | 7.8[c] |
| *Site of first cancer*: Corpus uteri | | | | |
| Any site* | | | 41 | 1.0 |
| Colorectum | | | 11 | 1.7 |
| Breast | | | 15 | 1.3 |
| *Site of first cancer*: Ovary | | | | |
| Any site* | | | 15 | 1.4 |
| Stomach | | | 3 | 7.6[b] |
| Colorectum | | | 2 | 1.2 |
| Breast | | | 5 | 1.5 |
| Corpus uteri | | | 2 | 2.7 |
| *Site of first cancer*: Prostate | | | | |
| Any site* | 91 | 0.7 | | |
| Colorectum | 18 | 1.0 | | |
| Stomach | 10 | 1.3 | | |
| Lung | 18 | 0.8 | | |
| Skin non-melanoma | 29 | 0.8 | | |
| Bladder | 11 | 1.5 | | |
| Kidney | 3 | 1.1 | | |
| Lymphomas | 6 | 1.5 | | |
| Leukaemias | 6 | 1.9 | | |

*continued*

**Table II** – *continued*

| *Site of second primary tumour* | *Males* Observed | SIR | *Females* Observed | SIR |
|---|---|---|---|---|
| *Site of first cancer*: Bladder | | | | |
| Any site* | 32 | 1.1 | 5 | 0.9 |
| Lung | 9 | 1.6 | 0 | – |
| Prostate | 10 | 1.8 | | |
| Kidney | 4 | 5.8[b] | 2 | 19.6[b] |
| *Site of first cancer*: Kidney | | | | |
| Any site* | 16 | 1.2 | 6 | 1.2 |
| Skin non-melanoma | 4 | 1.1 | 4 | 2.8 |
| Prostate | 4 | 1.7 | | |
| Bladder | 5 | 7.2[c] | 3 | 30.0[c] |
| *Site of first cancer*: Lymphomas | | | | |
| Any site* | 29 | 1.3 | 12 | 1.0 |
| Lung | 12 | 2.8[b] | 1 | 2.1 |
| Skin non-melanoma | 11 | 1.8[a] | 12 | 3.6[c] |
| *Site of first cancer*: Leukaemias | | | | |
| Any site* | 18 | 1.7[a] | 12 | 2.5[b] |
| Lung | 6 | 3.0[a] | 0 | – |
| Skin non-melanoma | 9 | 3.0[b] | 4 | 2.9[a] |

*Non-melanoma skin cancer excluded. [a]: $P<0.05$; [b]: $P<0.01$; [c]: $P<0.001$.

(SIR = 1.7 for males, 1.5 for females), non-melanomatous skin cancers (SIR = 1.6 for males, 1.5 for females), female breast (SIR = 1.3), kidney (SIR = 1.5 for males, 1.9 for females), and thyroid in males (SIR = 2.4).

Table II considers the incidence of selected subsequent primary neoplasms following each separate cancer site. The SIR following a cancer of the oral cavity and pharynx was around 3 in both sexes (based on 95 males and 20 females), and was mainly due to a substantial excess of subsequent primaries in the oral cavity (SIR = 13 for males), oesophagus (SIR around 20 for both sexes), larynx and lung (SIR over 6 for males). The incidence of subsequent primary neoplasms was only slightly above unity following oesophageal cancer, also on account of the extremely low survival rates, and hence limited man-years at risk, from this neoplasm. In males, this excess was due to cancers of the oral cavity and pharynx (significant) and lung. A total of 55 males had a subsequent primary following laryngeal cancer. The overall SIR for all neoplasms was 3.0, and significant excesses were observed for oral cavity or pharynx (SIR = 7.6), oesophagus (SIR = 5.3) and lung (SIR = 5.7). The number of second primaries following lung cancer was 72 among males (SIR = 1.7) and 12 among females (SIR = 2.6). In both sexes, a significantly elevated SIR was observed for cancers of the oral cavity and lung, and in males of the oesophagus, too.

With reference to second primaries following cancers of the stomach and colorectum, no overall excess was observed for all sites, but elevated SIRs were apparent for skin non-melanoma following gastric cancer, and for large bowel and prostatic cancer following colorectum.

A total of 16 males and 13 females experienced a second neoplasm following skin melanoma. Significant excess ratios were observed for colorectum in males (SIR = 2.6) and skin non-melanoma in females (SIR = 4.1). The numbers of second tumours were much larger for non-melanomatous skin cancers (336 males, 189 females), but the SIR for all sites was exactly 1.0 in both sexes. Significantly elevated SIRs were, however, observed for skin melanoma (SIR = 3.0 in males, 2.3 in females) and non-melanoma (SIR around 2 in both sexes).

With reference to second primaries following breast and female genital tract neoplasms, the incidence of any cancer following breast cancer was significantly elevated (SIR = 1.2), mainly on account of an elevated risk of subsequent breast cancer (SIR = 1.7). With reference to cervical cancer, the SIR was significantly elevated for any subsequent primary (SIR = 1.6) as well as for lung cancer (SIR = 7.8, based on seven cases). No overall excess of second primaries was observed after endometrial and ovarian cancer, although the incidence was (non-significantly) elevated for colorectal and breast cancers.

With respect to selected second primaries following urinary tract (bladder and kidney) and prostate cancer, significantly elevated SIRs were observed for kidney following bladder cancer (SIR = 5.8 in males and 19.6 in females, based on 4 and 2 cases, respectively), and for bladder following kidney (SIR = 7.2 in males and 30.0 in females, based on 5 and 3 cases, respectively). A total of 91 second primary neoplasms were registered among first occurring prostate neoplasms. No significant excess risk was observed, although SIR were above unity for stomach (SIR = 1.3), bladder (SIR = 1.5), lymphomas (SIR = 1.5) and leukaemias (SIR = 1.9).

Finally, second primaries were considered following leukaemias and lymphomas. In both sexes, the incidence of cancers of any site was significantly elevated following leukaemia (SIR = 1.77 in males, 2.5 in females), with a significant excess for lung in males and of non-melanomatous skin cancers in both sexes. Likewise, following lymphomas, there was an excess of lung cancers in males (SIR = 2.8), and of non-melanomatous skin in both sexes.

### Discussion

The present work has mainly a descriptive value, and provides further quantification on a population-based dataset of the subsequent risk of various (second metachronous) primary cancers following any specific neoplasm. Its main value lies in the fact that only a few similar series have been published (Curtis *et al.*, 1985; Storm *et al.*, 1985; Teppo *et al.*, 1985), and hence the contribution of this study, at least in quantitative terms of risk assessment, can be relevant. A further interest of the present dataset derives from the strict criteria adopted for the definition of second primaries, and the practically total histological confirmation and revision (Levi *et al.*, 1982, 1987). An important limitation of the study, however, is due to the relatively limited size of the present study population and of the length of the follow-up, and hence of the total number of multiple primary cancers examined, at least in comparison with similar studies from Connecticut (Curtis *et al.*, 1985), Denmark (Storm *et al.*, 1985), or Finland (Teppo *et al.*, 1985).

Another potential limitation of this study design is related to problems of registration of second cancers. Although these problems are reduced by an active follow-up of all registered cases (Levi *et al.*, 1989*b*), registration may be incomplete for some site, such as non-melanoma skin cancers. Therefore, non-melanoma skin cancer was excluded from the total number of second primaries following each separate first primary (Table II). Still, even for skin cancer registration this is a privileged and particularly well surveilled population (Levi *et al.*, 1988*a*). Some caution in the interpretation is also important, since over 1,000 comparisons were made, and hence some significant results are bound to occur by change alone.

Some of the associations emerged can be related to common risk factor exposure. For instance, the generally elevated risk of multiple primaries of the upper digestive and respiratory tract should be related to tobacco and alcohol consumption (Tuyns *et al.*, 1977; Franceschi *et al.*, 1990), and viewed against the baseline high rates of these neoplasms in French-speaking Switzerland (Levi *et al.*, 1989*a*). Tobacco consumption may also explain the excess of these neoplasms following lung cancer, and the elevated incidence of lung cancer following bladder and (probably) cervical cancer (US Department of Health and Human Services, 1982; IARC, 1986), although it is not obvious to explain, on this basis alone, the elevated leukaemia risk (Kabat *et al.*, 1988; Kinlen & Rogot, 1988).

In some instances tumours with different aetiologies may simply appear significantly associated because they share similar social class correlates. Along this line, the excesses of cancer of the colorectum and prostate subsequent to a diagnosis of skin melanoma, may be interpreted, at least in part, in terms of the more elevated prevalence of these three neoplasms in the highest social classes in Switzerland (Levi *et al.*, 1988*b*).

Other associations are well known, although quantification, again, may be of some interest. For instance, the risk of metachronous second primary breast cancer was elevated by 70% on a population (publick health) level, but the real excess risk for epidemiological and etiological inference is probably double, since most women have only one breast at risk following surgery for breast cancer (Peto, 1987). The excess breast cancer incidence following primary ovarian cancer may be due to genetic susceptibility (Parazzini *et al.*, 1992), besides common aetiological correlates (Franceschi, 1989). The association between multiple colorectal cancers, kidney and bladder cancer and melanoma and non-melanomatous skin cancers may be due to the action of common risk factor exposure, but also to the increased surveillance following a cancer diagnosis, which may be of particular relevance for skin neoplasms and explain the association of non- melanomatous skin cancer with other neoplasms (e.g., leukaemias).

In general, and in conclusion, the overall excess risk of a second primary cancer is relatively limited (about 20% in both sexes), and at least in part attributable to increased surveillance. Thus, the true excess risk, due to risk factor exposure or the consequences of treatment of the first neoplasm is even lower and, on a population level, of limited public health importance for most cancer sites. Noteworthy exceptions are represented by tumours of the upper aerodigestive tract where more than 5-to-10 fold increased cancer risks are common and clearly would justify special preventive and therapeutical efforts.

The authors wish to thank the Vaud Cancer Registry's staff (Mrs N. Menoud, Ms G. Kaufmann, Ms N. Puenzieux and Mrs G. Descombaz) to whom most of its results and accomplishments are due. The contribution of the Swiss League against cancer is gratefully acknowledged.

### References

BERG, J.W. (1967). The incidence of multiple primary cancers. I. Development of further cancers in patients with lymphomas, leukemias, and myeloma. *J. Natl Cancer Inst.*, **38**, 741–752.

BERG, J.W., SCHOTTENFELD, D. & RITTER, F. (1970). Incidence of multiple primary cancers. III. Cancers of the respiratory tract and upper digestive system as multiple primary cancers. *J. Natl Cancer Inst.*, **44**, 263–274.

BOICE, J.D., DAY, N.E., ANDERSEN, A., BRINTON, L.A., CHOI, N.W., CLARKE, E.A., COLEMAN, M.P., CURTIS, R.E., FLANNERY, J.T., HAKAMA, M., HAKULINEN, T., HOWE, G.R., JENSEN, O.M., KLEINERMAN, R.A., MAGNIN, D., MAGNUS, K., MAKELA, K., MALKER, B., MILLER, A.B., NELSON, N., PATTERSON, C.C., PETTERSON, F., POMPE-KIRN, V., PRIMIC-ZAKELI, M., PRIOR, P., RAVNIHAR, B., SKEET, R.G., SKJERVEN, J.E., SMITH, P.G., SOK, M., SPENGLER, R.F., STORM, H.H., STOVALL, M., TOMKINS, G.W.O. & WALL, C. (1985*a*). Second cancers following radiation treatment for cervical cancer. An international collaboration among cancer registries. *JNCI*, **74**, 955–975.

BOICE, J.D., STORM, H.H., CURTIS, R.E., JENSEN, O.M., KLEINERMAN, R.A., JENSEN, H.S.,FLANNERY, J.T. & FRAUMENI, J.F.Jr. (1985*b*). Introduction to the study of multiple primary cancers. *Natl Cancer Inst. Monogr.*, **68**, 3–9.

BOIVIN, J.F., HUTCHISON, G.B., EVANS, F.B., ABOU-DAOUD, K.T. & JUNOD, B. (1986).Leukemia after radiotherapy for first primary cancers of various anatomic sites. *Am. J. Epidemiol.*, **123**, 993–1003.

BRESLOW, N.E. & DAY, N.E. (1987). *Statistical methods in cancer research. The analysis of case-control studies.* Vol. 2. IARC Scient. Publ. 82: Lyon.

CURTIS, R.E., BOICE, J.D., KLEINERMAN, R.A., FLANNERY, J.T. & FRAUMENI, J.Jr. (1985). Summary: multiple primary cancers in Connecticut, 1935–82. *Natl Cancer Inst. Monogr.*, **68**, 219–242.

FRANCESCHI, S. (1989). Reproductive factors and cancer of the breast, ovary and endometrium. *Europ. J. Cancer Clin. Oncol.*, **25**, 1933–1943.

FRANCESCHI, S., TALAMINI, R., BARRA, S., BARÒN, A.E., NEGRI, E., BIDOLI, E., SERRAINO, D. & LA VECCHIA, C. (1990). Smoking and drinking in relation to cancers of the oral cavity, pharynx, larynx and esophagus in Northern Italy. *Cancer Res.*, **50**, 6502–6507.

IARC MONOGRAPH ON THE EVALUATION OF THE CARCINOGENIC RISK OF CHEMICALS TO HUMANS. (1986). Vol. 38. *Tobacco smoking.* IARC: Lyon.

KABAT, G.C., AUGUSTINE, A. & HEBERT, J.R. (1988). Smoking and adult leukemia: a case-control study. *J. Clin. Epidemiol.*, **41**, 907–914.

KALDOR, J.M., DAY, N.E., BAND, P., CHOI, N.W., CLARKE, E.A., COLEMAN, P., HAKAMA, M., KOCH, M., LANGMARK, F., NEAL, F.E., PETTERSON, F., POMPEKIRN, V., PRIOR, P. & STORM, H.H. (1987). Second malignancies following testicular cancer, ovarian cancer and Hodgkin's disease: an international collaborative study among cancer registries. *Int. J. Cancer*, **39**, 571–585.

KALDOR, J.M., DAY, N.E., PETTERSON, F., CLARKE, F.A., PEDERSEN, D., MEHNERT, W., BELL, J., HOST, H., PRIOR, P., KARJALAINEN, S., NEAL, F., KOCH, M., BAND, P., CHOI, W., POMPEKIRN, V., ZAREN, A.A.B., BELCH, A.R., STORM, H.H., KITTELMANN, B., FRASER, P. & STOVALL, M. (1990*a*). Leukemia following chemotherapy for ovarian cancer. *N. Engl. J. Med.*, **322**, 1–6.

KALDOR, J.M., DAY, N.E., CLARKE, F.A., VAN LEEUWEN, F.E., HENRY-AMAR, M., FIORENTINO, M.V., BELL, J., PEDERSEN, D., BAND, P., ASSOULINE, D., KOCH, M., CHOI, W., PRIOR, P., BLAIR, V., LANGMARK, F., POMPEKIRN, V., NEAL, F., PETERS, D., PFEIFFER, R., KARJALAINEN, S., CUZICK, J., SUTCLIFF, S.B., SOMERS, R., PELLAE-COSSET, B., PAPPAGALLO, G.L., FRASER, P., STORM, H. & STOVALL, M. (1990*b*). Leukemia following Hodgkin's disease. *N. Engl. J. Med.*, **322**, 7–13.

KINLEN, L.J. & ROGOT, E. (1988). Leukemia and smoking habits among United States Veterans. *Br. J. Med.*, **297**, 657–659.

LEVI, F. (1982). Statistics from the registry of the canton of Vaud, Switzerland, 1975–1977. In *Cancer Incidence in Five Continents.* Waterhouse, J., Muir, C.S., Shanmugaratnam, K., Powell, J., Peacham, D. & Whelan, S. (eds), Vol. IV. pp. 546–549. IARC Scient. Publ. 42: Lyon.

LEVI, F. (1987). Statistics from the registry of the canton of Vaud, Switzerland, 1978–1982. In *Cancer Incidence in Five Continents.* Muir, C.S., Waterhouse, J., Mack, T., Powell, J. & Whelan, S. (eds), Vol. V. pp. 634–639. IARC Scient. Publ. 88: Lyon.

LEVI, F., LA VECCHIA, C., TE, V.C. & MEZZANOTTE, G. (1988*a*). Descriptive epidemiology of skin cancer in the Swiss Canton of Vaud. *Int. J. Cancer*, **42**, 811–816.

LEVI, F., NEGRI, E., LA VECCHIA, C. & TE, V.C. (1988*b*). Socioeconomic groups and cancer risk at death in the Swiss Canton of Vaud. *Int. J. Epidemiol.*, **17**, 711–717.

LEVI, F., MAISONNEUVE, P., FILIBERTI, R., LA VECCHIA, C. & BOYLE, P. (1989*a*). Cancer incidence and mortality in Europe. *Soz. Praeventivmed.*, **34** (suppl. 2), S3–S84.

LEVI, F., MEZZANOTTE, G., TE, V.C. & LA VECCHIA, C. (1989*b*). Cancer survival from the incident cases of the Registry of Vaud, Switzerland. *Tumori*, **75**, 83–89.

PARAZZINI, F., NEGRI, E., LA VECCHIA, C., RESTELLI, C., FRANCESCHI, S. (1992). Family history of reproductive cancers and ovarian cancer risk: an Italian case-control study. *Am. J. Epidemiol.*, **135**, 35–40.

PETO, R. (1987). Only one breast remains at risk of contralateral cancer. *Brit. J. Cancer*, **55**, 352.

SCHOTTENFELD, D., BERG, J.W. & VITSKY, B. (1969). Incidence of multiple primary cancers. II. Index cancers arising in the stomach and lower digestive system. *J. Natl Cancer Inst.*, **43**, 77–86.

SCHOTTENFELD, D. & BERG, J. (1971). Incidence of multiple primary cancers. IV. Cancers of the female breast and genital organs. *J. Natl Cancer Inst.*, **46**, 161–170.

STORM, H.H., JENSEN, O.M., EWERTZ, M., LYNGE, E., OLSEN, J.H., SCOU, G. & OSTERLIND, A. (1985). Summary: multiple primary cancers in Denmark, 1943–80. *Natl Cancer Inst. Monogr.*, **68**, 411–430.

TEPPO, L., PUKKALA, E. & SAXEN, E. (1985). Multiple cancer – an epidemiologic exercise in Finland. *JNCI*, **75**, 207–211.

TUYNS, A.J., PEQUIGNOT, G. & JENSEN, O.M. (1977). Le cancer de l'oesophage en Ille-et-Vilaine en fonction du niveau de consommation d'alcool et de tabac. Des risques qui se multiplient. *Bull. Cancer*, **64**, 45–60.

US DEPARTMENT OF HEALTH AND HUMAN SERVICES. (1982). *The Health Consequences of Smoking: Cancer*. A Report of the Surgeon General of the Public Health Service, U.S. G.P.O: Washington.

US DEPARTMENT OF HEALTH AND HUMAN SERVICES. (1985). Multiple Primary Cancers in Connecticut and Denmark. *NCI Monograph* 68: Bethesda, MD.

WATANABE, S., KODAMA, T., SHIMOSATO, Y., ARIMOTO, H., SUGIMURA, T., SUEMASU, K. & SHIRAISI, M. (1984). Multiple cancers in 5,456 autopsy cases in the National Cancer Center of Japan. *JNCI*, **72**, 1021–1027.


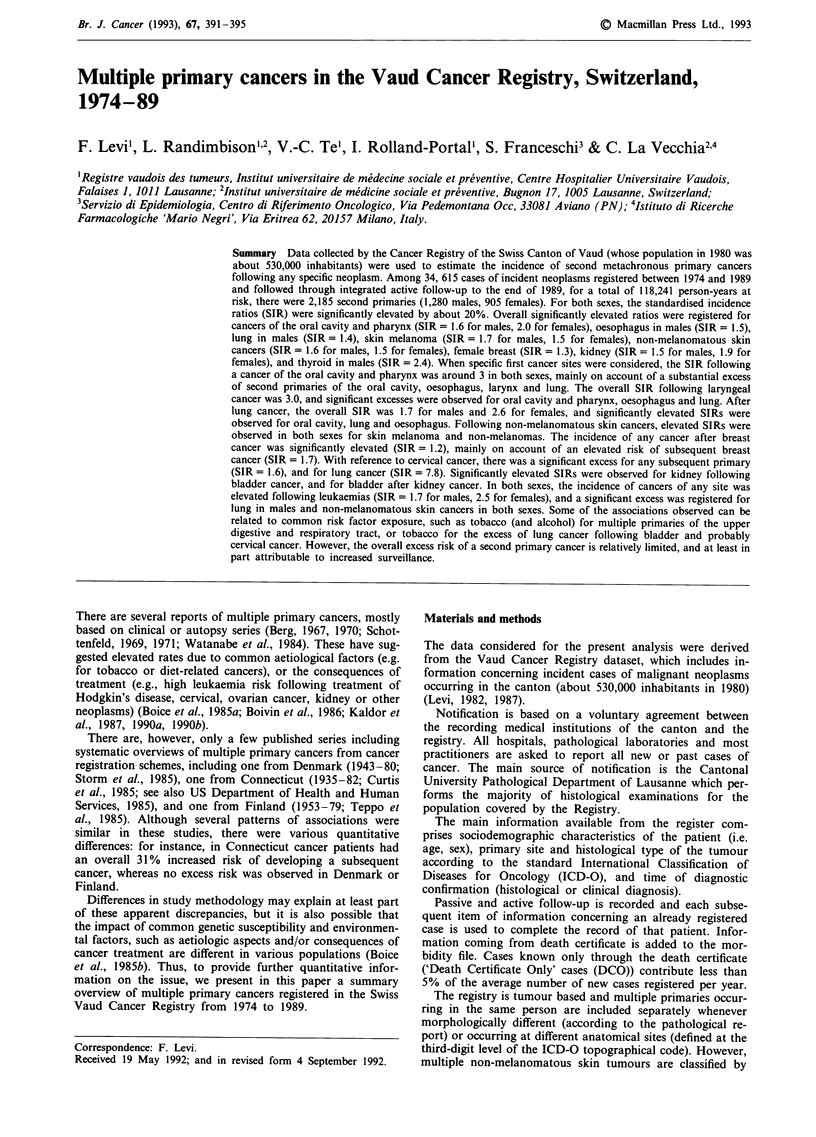

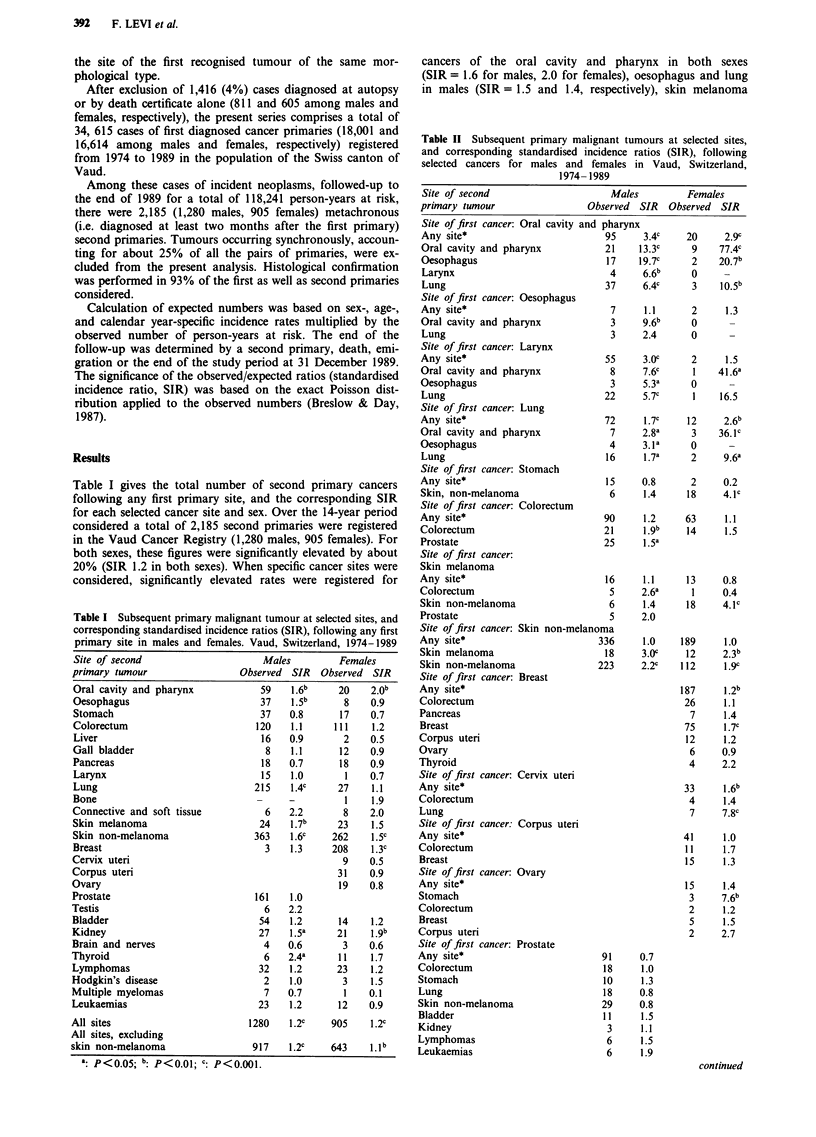

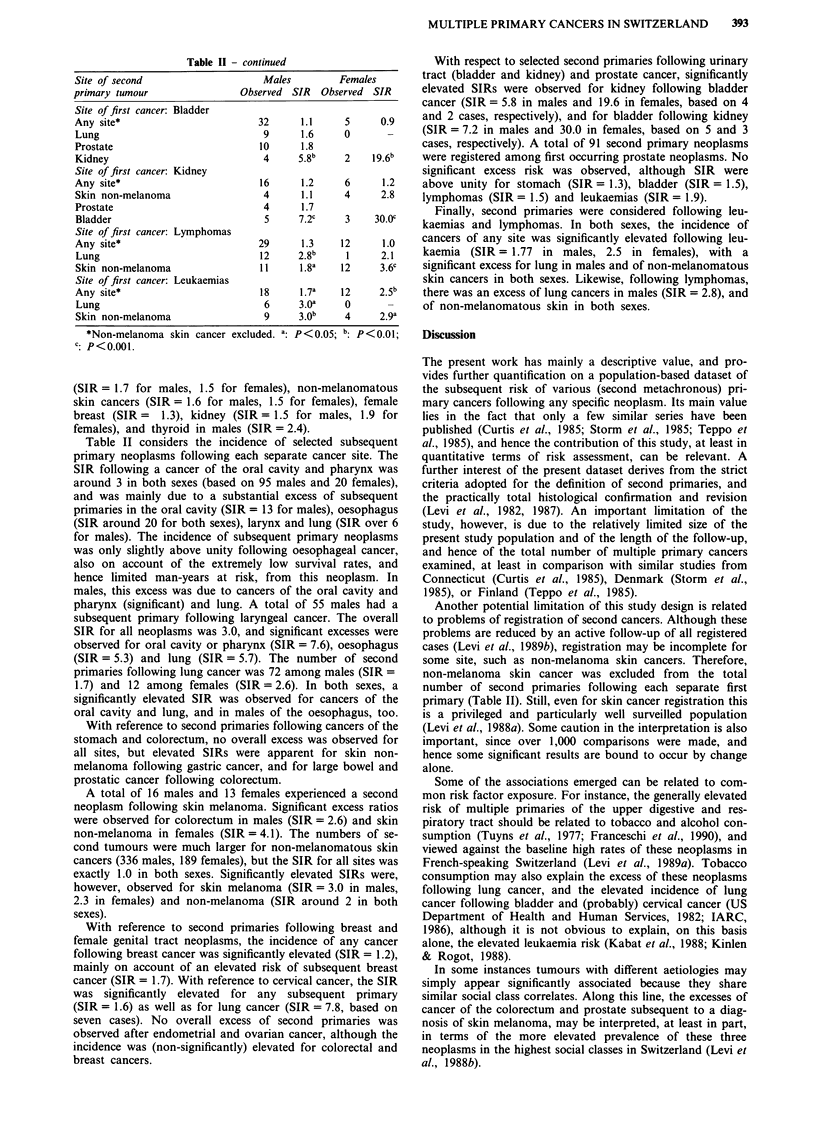

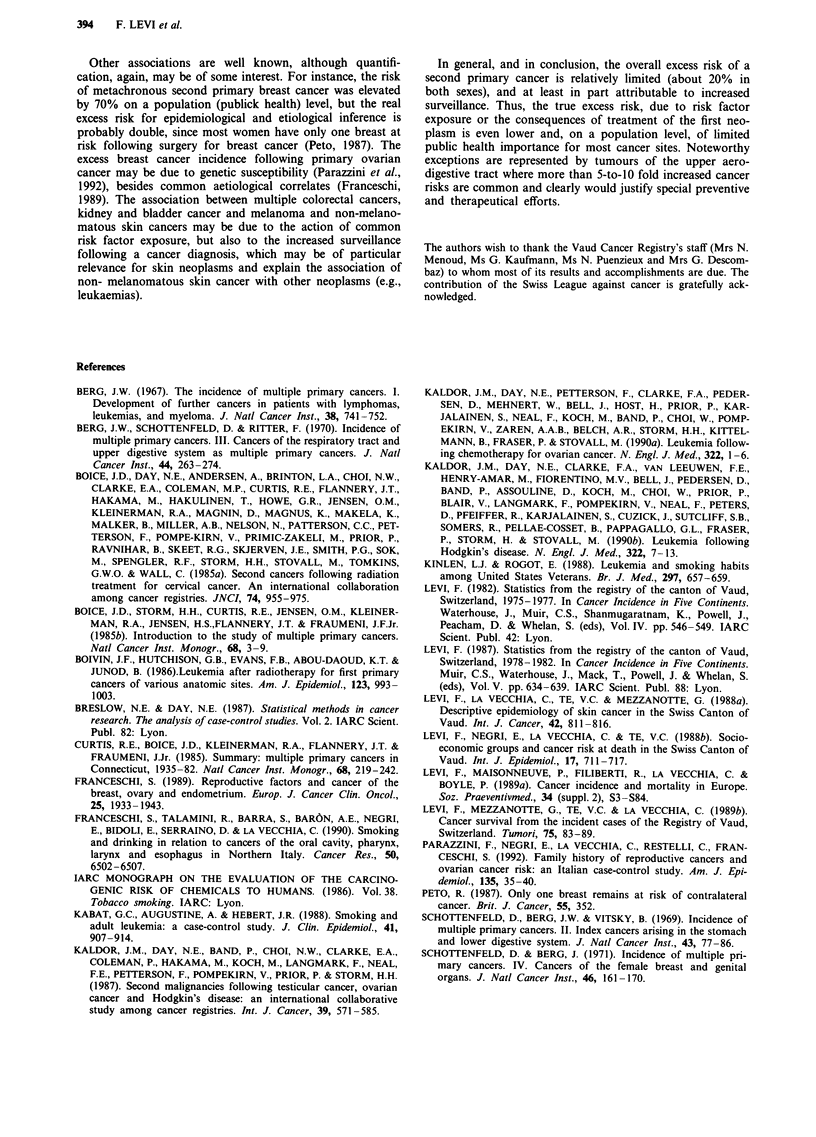

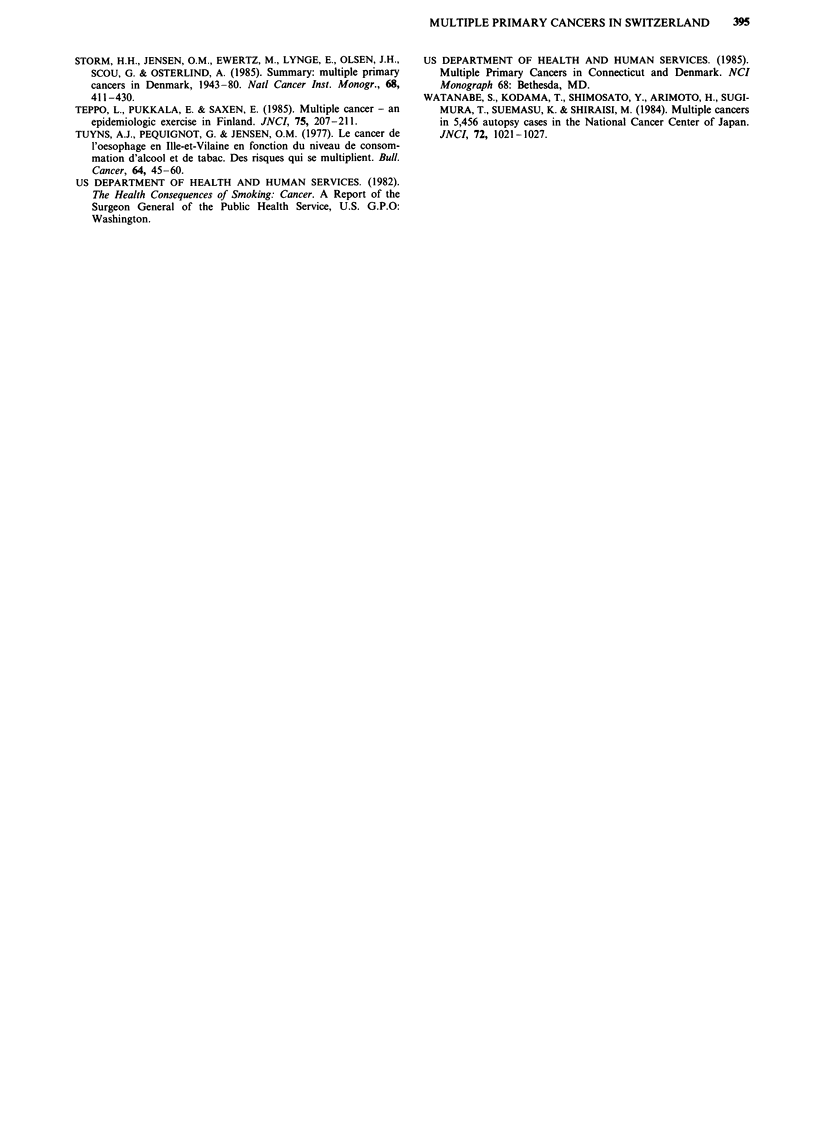


## References

[OCR_00667] Berg J. W., Schottenfeld D., Ritter F. (1970). Incidence of multiple primary cancers. III. Cancers of the respiratory and upper digestive system as multiple primary cancers.. J Natl Cancer Inst.

[OCR_00662] Berg J. W. (1967). The incidence of multiple primary cancers. I. Development of further cancers in patients with lymphomas, leukemias, and myeloma.. J Natl Cancer Inst.

[OCR_00679] Boice J. D., Day N. E., Andersen A., Brinton L. A., Brown R., Choi N. W., Clarke E. A., Coleman M. P., Curtis R. E., Flannery J. T. (1985). Second cancers following radiation treatment for cervical cancer. An international collaboration among cancer registries.. J Natl Cancer Inst.

[OCR_00692] Boivin J. F., Hutchison G. B., Evans F. B., Abou-Daoud K. T., Junod B. (1986). Leukemia after radiotherapy for first primary cancers of various anatomic sites.. Am J Epidemiol.

[OCR_00703] Curtis R. E., Boice J. D., Kleinerman R. A., Flannery J. T., Fraumeni J. F. (1985). Summary: multiple primary cancers in Connecticut, 1935-82.. Natl Cancer Inst Monogr.

[OCR_00707] Franceschi S. (1989). Reproductive factors and cancers of the breast, ovary and endometrium.. Eur J Cancer Clin Oncol.

[OCR_00712] Franceschi S., Talamini R., Barra S., Barón A. E., Negri E., Bidoli E., Serraino D., La Vecchia C. (1990). Smoking and drinking in relation to cancers of the oral cavity, pharynx, larynx, and esophagus in northern Italy.. Cancer Res.

[OCR_00724] Kabat G. C., Augustine A., Hebert J. R. (1988). Smoking and adult leukemia: a case-control study.. J Clin Epidemiol.

[OCR_00729] Kaldor J. M., Day N. E., Band P., Choi N. W., Clarke E. A., Coleman M. P., Hakama M., Koch M., Langmark F., Neal F. E. (1987). Second malignancies following testicular cancer, ovarian cancer and Hodgkin's disease: an international collaborative study among cancer registries.. Int J Cancer.

[OCR_00744] Kaldor J. M., Day N. E., Clarke E. A., Van Leeuwen F. E., Henry-Amar M., Fiorentino M. V., Bell J., Pedersen D., Band P., Assouline D. (1990). Leukemia following Hodgkin's disease.. N Engl J Med.

[OCR_00742] Kaldor J. M., Day N. E., Pettersson F., Clarke E. A., Pedersen D., Mehnert W., Bell J., Høst H., Prior P., Karjalainen S. (1990). Leukemia following chemotherapy for ovarian cancer.. N Engl J Med.

[OCR_00754] Kinlen L. J., Rogot E. (1988). Leukaemia and smoking habits among United States veterans.. BMJ.

[OCR_00771] Levi F., La Vecchia C., Te V. C., Mezzanotte G. (1988). Descriptive epidemiology of skin cancer in the Swiss Canton of Vaud.. Int J Cancer.

[OCR_00781] Levi F., Maisonneuve P., Filiberti R., La Vecchia C., Boyle P. (1989). Cancer incidence and mortality in Europe.. Soz Praventivmed.

[OCR_00786] Levi F., Mezzanotte G., VAN CONG T. E., La Vecchia C. (1989). Cancer survival from the incident cases of the Registry of Vaud, Switzerland.. Tumori.

[OCR_00776] Levi F., Negri E., La Vecchia C., Te V. C. (1988). Socioeconomic groups and cancer risk at death in the Swiss Canton of Vaud.. Int J Epidemiol.

[OCR_00793] Parazzini F., Negri E., La Vecchia C., Restelli C., Franceschi S. (1992). Family history of reproductive cancers and ovarian cancer risk: an Italian case-control study.. Am J Epidemiol.

[OCR_00801] Schottenfeld D., Berg J. W., Vitsky B. (1969). Incidence of multiple primary cancers. II. Index cancers arising in the stomach and lower digestive system.. J Natl Cancer Inst.

[OCR_00806] Schottenfeld D., Berg J. (1971). Incidence of miltiple primary cancers. IV. Cancers of the female breast and genital organs.. J Natl Cancer Inst.

[OCR_00813] Storm H. H., Jensen O. M., Ewertz M., Lynge E., Olsen J. H., Schou G., Osterlind A. (1985). Summary: multiple primary cancers in Denmark, 1943-80.. Natl Cancer Inst Monogr.

[OCR_00819] Teppo L., Pukkala E., Saxén E. (1985). Multiple cancer--an epidemiologic exercise in Finland.. J Natl Cancer Inst.

[OCR_00823] Tuyns A. J., Péquignot G., Jensen O. M. (1977). Le cancer de l'oesophage en Ille-et-Vilaine en fonction des niveaux de consommation d'alcool et de tabac. Des risques qui se multiplient. Bull Cancer.

[OCR_00842] Watanabe S., Kodama T., Shimosato Y., Arimoto H., Sugimura T., Suemasu K., Shiraishi M. (1984). Multiple primary cancers in 5,456 autopsy cases in the National Cancer Center of Japan.. J Natl Cancer Inst.

